# Neutrophil Migration in the Activation of the Innate Immune Response to Different *Flavobacterium psychrophilum* Vaccines in Zebrafish (*Danio rerio*)

**DOI:** 10.1155/2015/515187

**Published:** 2015-02-28

**Authors:** Camila J. Solís, Matías Poblete-Morales, Sergio Cabral, Juan A. Valdés, Ariel E. Reyes, Ruben Avendaño-Herrera, Carmen G. Feijóo

**Affiliations:** ^1^Departamento de Ciencias Biologicas, Facultad de Ciencias Biologicas, Universidad Andres Bello, 8370146 Santiago, Chile; ^2^Interdisciplinary Center for Aquiculture Aquaculture Research, (INCAR), 8340518 Concepción, Chile; ^3^SEPPIC Brazil, Rua Libero Badaro 182, 01008000 São Paolo, SP, Brazil; ^4^Centro de Investigación Marina Quintay (CIMARQ), Quintay, Chile

## Abstract

*Flavobacterium psychrophilum* is a Gram-negative bacterium, responsible for the bacterial cold-water disease and the rainbow trout fry syndrome in freshwater salmonid fish. At present, there is only one commercial vaccine in Chile, made with two Chilean *F. psychrophilum* isolates and another licensed in Europe. The present study analyzed neutrophil migration, as a marker of innate immune activation, in zebrafish (*Danio rerio*) in response to different *F. psychrophilum* bath vaccines, which is the first step in evaluating vaccine effectiveness and efficiency in fish. Results indicated that bacterins of the LM-02-Fp isolate were more immunogenic than those from the LM-13-Fp isolate. However, no differences were observed between the same bacteria inactivated by either formaldehyde or heat. Importantly, the same vaccine formulation without an adjuvant only triggered a mild neutrophil migration compared to the complete vaccine. Observations also found that, after a year of storage at 4°C, the activation of the innate immune system by the different vaccines was considerably decreased. Finally, new vaccine formulations prepared with heat and formaldehyde inactivated LM-02-Fp were significantly more efficient than the available commercial vaccine in regard to stimulating the innate immune system.

## 1. Introduction

*Flavobacterium psychrophilum* [1], a Gram-negative, filamentous, psychrotrophic bacterium belonging to the phylum Bacteroidetes, is the causative agent of bacterial cold-water disease (BCWD) and rainbow trout fry syndrome (RTFS) in freshwater salmonid fish worldwide [2, 3]. In Chile, this infectious bacterium was first observed in freshwater aquaculture facilities in 1993, and the incidence of* F. psychrophilum* has dramatically increased since then [4, 5].

Despite the severe impact of this disease, antimicrobial therapies are currently the only method for controlling this condition in farmed fish, and it has been estimated that 55 tons of florfenicol and oxytetracycline each were used in Chilean farms to control outbreaks between 2006 and 2009 [6]. A tentatively licensed commercial vaccine that contains whole-cells inactivated with formaldehyde, Flavomune vaccine (SAG N°2160-BP), was recently developed in Chile. However it is important to note that its efficacy was tested using injection-based challenge models that completely bypassed the protective functions of the fish skin-mucus layer. This is significant due to this layer acting as an important barrier to disease infection in fish.

One key for efficient immunization and a powerful generation of specific antibodies is to ensure efficient antigen presentation by cells from the innate immune system. This event triggers an antigen-specific adaptive immune response that leads to the production of specific antibodies against an invading pathogen [7]. Activated, or noncirculating, leukocytes are involved in the initiation of the adaptive immune response [8]. Recent studies have moreover established various factors that elicit the innate immune response shaping adaptive immunity [8–13]. For instance, splenic neutrophils facilitate the antibody response of marginal zone B cells to microbial antigens [13]. These granulocytes promote B cell survival, as well as the production of IgM, IgG, and IgA antibodies [13, 14].

During the last decade, the zebrafish (*Danio rerio*) has been positioned as a powerful model for immunity research not only for other fish species, but also for mammals [15–19]. Some outstanding characteristics are its genetic tractability and transparency during the embryonic and larval stages, which facilitate monitoring infection and inflammation processes* in vivo* [18, 20–24]. Early during zebrafish development, the innate immune system exists in isolation to the adaptive system, which develops later in the larval stages and requires 4–6 weeks before achieving full functionality [25].

In the present work, zebrafish were used to compare the effect of different* F. psychrophilum* vaccines on the activation of the innate immune system, using neutrophils as specific innate immune system markers. These leukocytes are the first cells to be mobilized in response to injury, and they are the first to infiltrate damaged tissue [26–29]. For all assays, the* Tg(mpx:GFP)* ^*i114*^ transgenic zebrafish line was used given that it exclusively expresses the green fluorescent protein (GFP) in neutrophils and allows tracking individual immune cells in live animals [30]. Zebrafish larvae were bath-vaccinated with three strains of bacteria inactivated by heat (VAH) or formaldehyde (VAF) and with or without an adjuvant. Afterwards, neutrophil migration was determined to investigate the effects of vaccination on the activation of the innate immune system.

## 2. Materials and Methods

### 2.1. Selection of Bacterial Strains for Vaccine Formulation

Two* F. psychrophilum* strains (LM-02-Fp and LM-13-Fp) were isolated in 2006 in Chile from the kidneys of clinically infected rainbow trout (*Oncorhynchus mykiss*) for subsequent vaccine preparation. The LM-02-Fp isolate is a sequence type (ST) 2 strain and antigenically Group 1 (1150), while LM-13-Fp is a ST12 strain and Group 2 (1658) [31, 32]. The identity of each isolate was confirmed as* F. psychrophilum* by using standard phenotyping procedures [33], including analyses of colony morphology and pigmentation, cell morphology, gliding motility, Gram-staining, cytochrome oxidase and catalase activities, oxidation/fermentation reactions, the presence of cell wall-associated flexirubin-type pigments, and the absorption of Congo red. Each isolate was routinely grown on a tryptone yeast extract salts medium (TYES; 0.4% tryptone, 0.05% yeast extract, 0.02% anhydrous calcium chloride, and 0.05% magnesium sulphate heptahydrate; pH 7.2) in either a liquid or solid state (TYES supplemented with 1% (w/v) agar bacteriological). Bacteria were aerobically incubated at 15°C for 3–5 days. Stock cultures were then frozen and kept at −80°C in Criobilles tubes (AES Laboratory) or in a TYES broth with 10% glycerol.

### 2.2. Vaccine Preparation

The selected* F. psychrophilum* isolates were grown in TYES broth for 56 h in order to obtain a final concentration of 3.49 ± 0.51 × 10^8^ colony forming units (CFU) mL^−1^. Eight vaccines (including* F. psychrophilum* cells and extracellular products) (Table 1) were prepared using the following two inactivation protocols: (i) LM-02-Fp and LM-13-Fp cultures were inactivated by heat at 60°C for 6 h and then stored at 4°C; and (ii) LM-02-Fp and LM-13-Fp cultures were inactivated through the addition of formalin to a final concentration of 0.7% and then stored at 4°C. The efficiency of each inactivation method was determined by spreading 0.1 mL of culture in 10-fold dilutions (10^−1^ to 10^−8^ in duplicate) onto a TYES agar plates. Subsequently, the presence of bacterial colonies was monitored for 5 days. Once the plate counts were negative for* F. psychrophilum*, 1 mL of the culture was taken directly from each inactivation treatment (VAH or VAF) and seeded onto TYES agar.

Aliquots from the VAF or VAH vaccines based on LM-02-Fp and LM-13-Fp, respectively, were mixed 1 : 1 with the adjuvant Montanide IMS 1312 VG PR (SEPPIC, France) according to the manufacturer's protocols. In addition to this, the commercial Flavomune vaccine (SAG N°2160.BP), made with two Chilean* F. psychrophilum* strains (coded 1 and 4), was included for comparative purposes. During the study, the commercial vaccine was kept at 4°C until use.

### 2.3. Zebrafish Strains and Maintenance

Zebrafish were maintained and raised at Universidad Andrés Bello facilities according to standard protocols [34]. The strain of zebrafish used was* Tg(mpx:GFP)* ^*i114*^ [30]. All embryos were collected by natural spawning, staged according to Kimmel and Colleagues [21], and raised in Petri dishes at 28.5°C in an E3 medium (5 mM of NaCl, 0.17 mM of KCl, 0.33 mM of CaCl_2_, 0.33 mM of MgSO_4_, and without methylene blue; pH 7.0) as previously described [35]. Embryonic and larval stadium were expressed in hours post-fertilization (hpf).

### 2.4. Larvae Vaccination and Quantification of Neutrophil Migration

**Figure 1:**
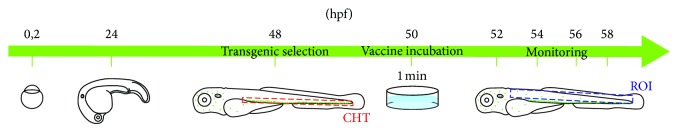
Scheme of vaccine assay. At 48 hpf* Tg(mpx:GFP)* ^*i114*^ nonactivated zebrafish larva, with neutrophils located only in the CHT (demarked in red), was selected, bath-incubated, and quantified the number of neutrophils mobilized to the region of interest (ROI, demarked in blue) at 2, 4, 6, and 8 hpi.

Fifteen* Tg(mpx:GFP)* ^*i114*^ larvae were selected at 48 hpf, as previously described [36], and bath-vaccinated by incubation with the different vaccines at a 1 : 10 dilution for 1 min (Table 1). A control medium with or without adjuvant was also included. After the bath-vaccination, larvae were washed three times for 5 min with the E3 medium, and neutrophils were monitored. Quantification of neutrophil migration was performed by analyzing the displacement of GFP positive cells outside of the caudal hematopoietic tissue (CHT) to the region of interest (ROI) (Figure 1) at 2, 4, 6, and 8 hours post-incubation (hpi) under a fluorescent stereoscope as previously described [36].

### 2.5. Imaging and Statistical Analyses

Photographs were taken with a QImaging MicroPublisher 5.0 RVT camera using the Olympus SZX16 stereoscope. Images were processed with Photoshop CS5.1, with pictures showing the representative effects of each treatment. Data were analyzed by two-way ANOVA using the Prism 6 software (GraphPad Software).

## 3. Results

**Figure 2:**
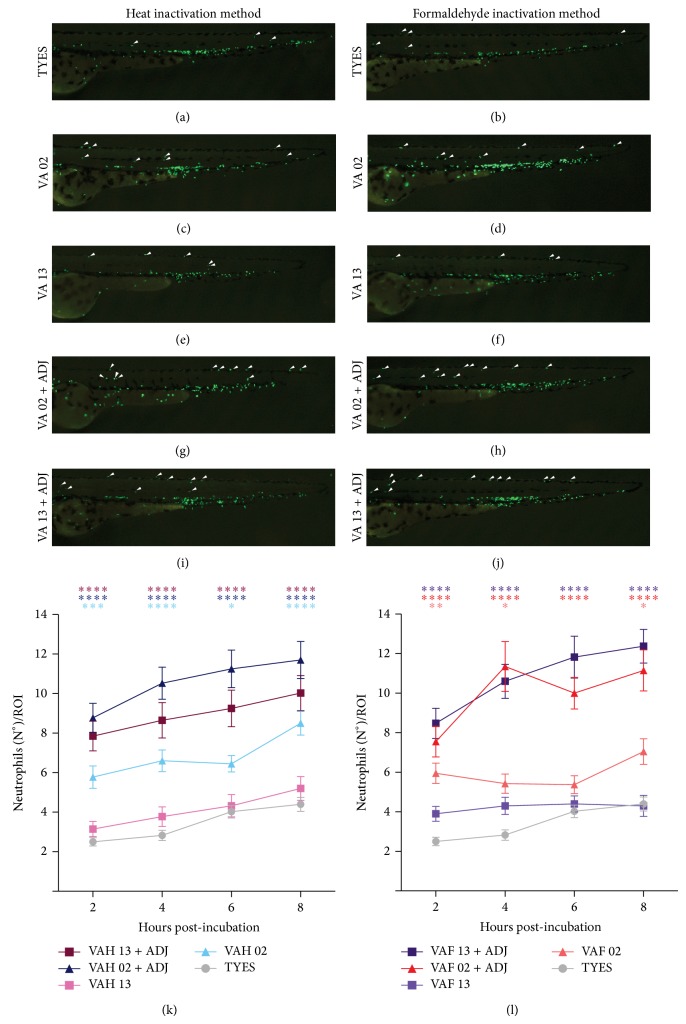
Effect on innate immune response triggered by freshly prepared vaccines against* F. psychrophilum*. ((a)–(j)) Representative images of zebrafish tails from larvae at 58 hpf. ((k) and (l)) Graph quantifying the number of neutrophils outside of the CHT (ROI), showing the effect on the innate immune response in larvae bath-vaccinated with (k) heat inactivated bacteria or (l) formaldehyde. Statistical analysis was performed using two-way ANOVA. All embryos ((a)–(j)) were oriented anterior to the left, with the dorsal on top. Data represent the mean ± standard error from three independent experiments with 15 larvae each. ^*^ *P* value < 0.05, ^***^ *P* < 0.001, and ^****^ *P* < 0.0001.

To analyze the innate immune response triggered by the different vaccine formulations, neutrophil behavior was analyzed in live zebrafish larvae (Figure 1). To quantify the number of mobilized neutrophils, a specific region in the larval tail was analyzed (ROI) since neutrophils are normally restricted to the CHT, with few circulating cells. Analysis of quantified neutrophil migration indicated that only VAH and VAF LM-02-Fp vaccines were able to trigger an innate immune response (Figures 2(c), 2(d), 2(k), and 2(l)). The number of neutrophils detected outside the CHT in larvae treated with the VAH or VAF LM-13-Fp vaccine was the same as that with the TYES medium, the negative control (Figures 2(e)–2(h), 2(k), and 2(l)). No statistical differences in the number of mobilized neutrophils were found between same-strain vaccines made from either heat or formaldehyde inactivation (Figures 2(k) and 2(l)). As was expected, when an adjuvant was added to the vaccine formulation all responses considerably increased, even in the TYES medium group (Figures 2(k) and 2(l); Figure S1 in Supplementary Material available online at http://dx.doi.org/10.1155/2015/515187). This result indicates that the adjuvant by itself was able to trigger the innate immune response.

**Figure 3:**
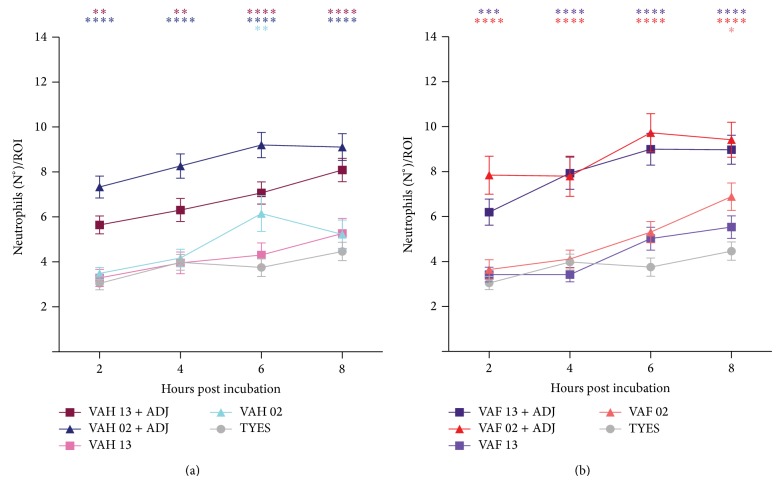
Effect on innate immune response triggered by one-year-old stored vaccines. ((a) and (b)) Graph quantifying neutrophil migration outside of the CHT (ROI), as triggered by bath-vaccination with formulas prepared from (a) heat or (b) formaldehyde inactivated bacteria. Statistical analysis was performed using two-way ANOVA. Data represent the mean ± standard error from three independent experiments with 15 larvae each. ^*^ *P* value < 0.05, ^**^ *P* < 0.005, ^***^ *P* < 0.001, and ^****^ *P* < 0.0001.

In order to determine if vaccine effects changed with time, the same assays as previously described were performed again using one-year-old vaccines. Results indicated that all vaccines had diminished activity (Figure 3). In the case of the VAH and VAF LM-02-Fp vaccines with an adjuvant, a freshly prepared vaccine mobilized an average of 11 neutrophils from the CHT at 8 hpi (Figures 2(k) and 2(l)). This is in contrast to the old vaccines, which moved approximately 8 neutrophils outside of the CHT after 8 hpi (Figure 3). Likewise, the one-year-old VAH and VAF LM-02-Fp vaccines without an adjuvant showed considerably diminished activity. Moreover, the old VAH LM-02-Fp vaccine only started to exert effects, as compared to the control, at 6 hpi, while the VAF LM-02-Fp vaccine showed effects at 8 hpi (Figure 3). These results suggest that a year of storage is too long of a time for maintaining efficacy.

**Figure 4:**
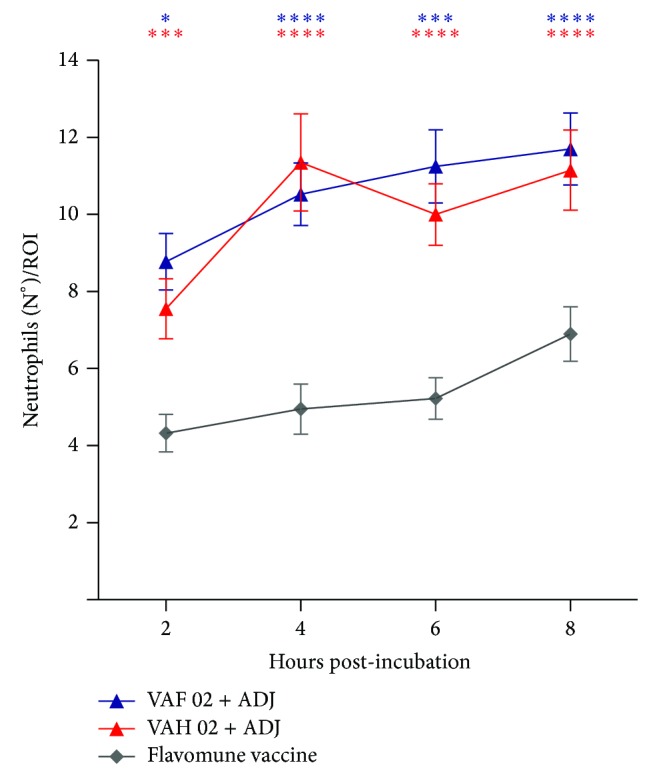
Comparison of the effects on the innate immune response between Flavomune vaccine and newly formulated vaccines. Graph quantifying neutrophil migration to the ROI for larvae bath-vaccinated with VAF-02-Fp + adjutant or VAH-02-Fp + adjutant, thus showing the activation of innate immune response. Statistical analysis was performed with a two-way ANOVA. Data represent the mean ± standard error from three independent experiments with 15 larvae each. ^*^ *P* value < 0.05, ^***^ *P* < 0.005, and ^****^ *P* < 0.0001.

Of significant note, however, is that both vaccines newly formulated with the isolate LM-02-Fp triggered an innate immune response at least twice as powerful as that produced by the commercial Flavomune vaccine (Figure 4).

## 4. Discussion

Vaccination is currently one of the most used practices in the prevention of fish diseases. Therefore, it is very important for the aquaculture industry to know the efficiency and effectiveness of the different commercially available vaccines. A preliminary step in selecting the most suitable vaccine against a specific pathogen would be to determine its effect on the innate immune system given that the activation of this system could yield a higher production of specific antibodies. In contrast, antigens unable to activate the innate immune response would probably induce a poor production of antibodies.

In Chile, autochthonous immersion bacterins made from single farm isolates have been used for years as protection against infection by* F. psychrophilum* [37], but there is still little information on how effective these antipathogen treatments are in terms of relative survival. Moreover, the currently available commercial vaccine against* F. psychrophilum* is insufficient, although there have been numerous studies towards developing a global commercial vaccine [38].

At the present time, there are no comparative studies in fish regarding the responses triggered by formaldehyde or heat inactivated antigens; only there studies using bath-vaccinations on fish from the same population. In fact, there are no concordant results in previous works performed with different vaccines containing formaldehyde- or heat-inactivated virulent cells of* F. psychrophilum*. This situation could be explained due to experiments being developed in different fish species (rainbow trout; Coho salmon (*Oncorhynchus kisutch*) and ayu (*Plecoglossus altivelis*)). Moreover, the vaccine formula used in previous studies differed in the content of adjuvant [39–45]. Importantly, Madetoja and Colleagues [46] compared the efficacy of formaldehyde- and heat-inactivated antigens in rainbow trout by intraperitoneal injection, demonstrating that both antigens produced high circulating antibody levels and efficient protection against the* F. psychrophilum.* The present results agree with this study, with LM-02-Fp and LM-13-Fp, regardless of the inactivation protocol, showing similar innate immune responses in zebrafish.

However, the current study used a set of vaccines supplemented with Montanide IMS 1312 VG, an aqueous adjuvant containing liquid particles, and an immunostimulating compound listed as a GRAS substance (generally recognized as safe). This adjuvant improves the presentation of the antigen to the immune cells and keeps it within the fish tissues, thus increasing the length of protection. It is important to highlight that it does not cause off-target effects such as melanosis [47]. In this sense, the present results demonstrate that Montanide IMS 1312 VG can be used in studies on fish vaccination against* F. psychrophilum*. Moreover, this adjuvant is able to activate an innate immune response. At present, further immunological studies are in progress in rainbow trout in order to establish the adaptive immune responses to specific antigens (unpublished data).

Another factor that should be studied in depth before designing an effective vaccine is the serotyping within the bacterial species. Mata and Colleagues [48] proposed a serotyping system for* F. psychrophilum* using slide agglutination and compared it to the serological schemes previously proposed by Lorenzen and Olesen [49] and Izumi and Wakabayashi [50]. Three to seven serotypes, mainly associated with the host species, were established [47]. In Chile, Valdebenito and Avendaño-Herrera [51] determined the existence of antigenic heterogeneity within* F. psychrophilum* with four patterns of serological reactions. According to this serological scheme, LM-02-Fp corresponds to the Group 1, while the strain LM-13-Fp belongs to the minor serological Group 4. Thus, a possible explanation for the differences in the results obtained for LM-02-Fp and LM-13-Fp on the innate immune response in zebrafish may be the existence of different antigenic group. Therefore, differences in serotype should be determined, and cross protection experiments have to be carried out in order to design a vaccine with broad-spectrum effectiveness.

In order to compare effects on the activation of the innate immune system for newly formulated vaccines (VAH and VAF LM-02-Fp) and the commercial Flavomune vaccine, the aforementioned assay was repeated and found that both newly formulated vaccines were largely more immunogenic than Flavomune vaccine. It is important to note that this commercial vaccine contains whole-cells from two Chilean* F. psychrophilum* strains inactivated by formaldehyde incubation (http://www.veterquimica.cl/contenido/vacuna-flavomune548).

The assay method applied in the present study permitted the analysis of the initial, yet essential, stage of efficient vaccine selection, which is the activation of cellular machinery that will promote antigen presentation and the innate immune response [32]. Results showed that both vaccines with adjuvants (strain LM-02-Fp and LM-13-Fp) exerted similar effects on the innate immune response, but only the LM-02-Fp strain was able to induce neutrophil migration without an adjuvant. From this, it can be speculated that the VAH/VAF LM-02-Fp vaccines could induce the generation of specific antibodies against* F. psychrophilum*. This isin contrast to theeffects triggered by VAH/VAF LM-13-Fp vaccines, which produced a nonspecific immune response that was mainly due to the adjuvant. These results suggest thatthe VAH and VAF LM-02-Fp vaccines would be a better candidate for vaccine formulation.

Finally, by using zebrafish as a model, another important outcome of this work was providing an in depth investigation on the mechanisms that control adaptive immunity in fish. Presently, fish vaccines are primarily developed according to mammalian knowledge about adaptive immunity, a scenario that might not be the most appropriate given the differences between fish and mammalian immune systems [52–55]. It is currently accepted that fish and particularly zebrafish share similarities with the mammalian immune system [19, 52, 56–58]. For example, both have a comparable set of immune cells [59–63], and the adaptive immune system is active later during embryonic development, after innate immune system development [25]. However, there are also differences as compared with mammalian immune responses, such as with the most abundant immunoglobulin in fish being IgM, whereas in mammals this is IgA [53, 64, 65]. Besides this, several important processes have not yet been described in fish, including antigen presentation and the existence of memory cells. In this regard, the different transgenic zebrafish lines with fluorescently labeled immune cells [30, 60, 61, 66–69] allow this teleost fish to act as a “live indicator” of immune processes, with the whole event of interest being monitored* in vivo*.

## 5. Conclusions

The VAH and VAF LM-02-Fp vaccine induced the strongest innate immune response, even when compared with the commercial Flavomune vaccine. However, after a year of storage at 4°C, the capacity of both formulated vaccines to trigger an innate immune response considerably decreased. Finally, the methodology described in this paper, using the zebrafish as fish model, is a suitable approach for analyzing mechanisms that control adaptive immunity in fish, as well pathways of bacterial infection and the innate immune response triggered by different vaccines.

## Supplementary Material

Supplementary Figure 1: Effect on the innate immune response triggered by control medium. Plot of quantified neutrophil migration to the ROI. Statistical analysis was performed by two-way ANOVA. Data represent the mean ± standard error from three independent experiments with 15 larvae each. ∗∗∗∗*P*-value <0.0001.

## Figures and Tables

**Table 1:** Vaccine formulation.

Strain	Inactivation method			
	Heat		Formaldehyde	
	Without adjuvant	With adjuvant	Without adjuvant	With adjuvant
LM-02-Fp	VAH 02	VAH 02 + Adj.	VAF 02	VAF 02 + Adj.
LM-13-Fp	VAH 13	VAH 13 + Adj.	VAF 13	VAF 13 + Adj.
Control medium	TYES (H)	TYES (H) + Adj.	TYES (F)	TYES (F) + Adj.
