# Supplementary figures and images for: Neutrophil Migration in the Activation of the Innate Immune Response to Different Flavobacterium psychrophilum Vaccines in Zebrafish (Danio rerio)
Source: J Immunol Res. 2015 Feb 28;2015:515187. doi: 10.1155/2015/515187 (PMC4359811; doi:10.1155/2015/515187)

0 hpf

24 hpf

48 hpf

50 hpf

52 hpf

54 hpf

56 hpf

58 hpf

*Transgenic Selection*

*Vaccine incubation*

*Monitoring*

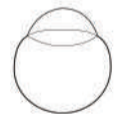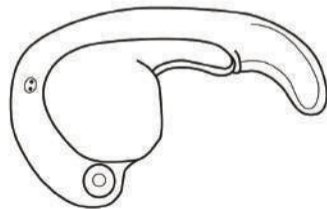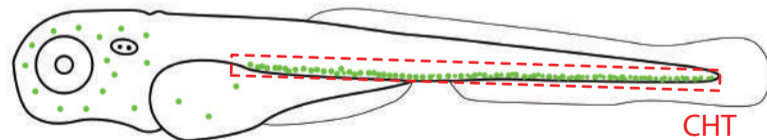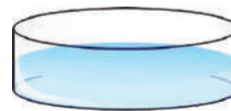

1 minute

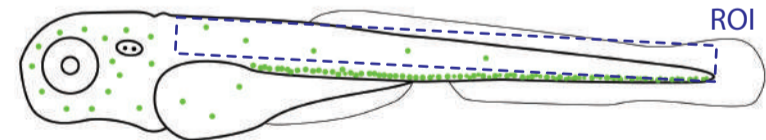

Supplement: Supplementary file 1 — Supplementary Figure 1: Effect on the innate immune response triggered by control medium. Plot of quantified neutrophil migration to the ROI. Statistical analysis was performed by two-way ANOVA. Data represent the mean ± standard error from three independent experiments with 15 larvae each. ∗∗∗∗P-value <0.0001. [file 515187.f1.pdf]
